# The Correlation of Hysteroscopy and Histopathology in Evaluating Abnormal Uterine Bleeding: Observations From a Tertiary Care Center

**DOI:** 10.7759/cureus.67807

**Published:** 2024-08-26

**Authors:** Pankaj Salvi, Sneha Aramandla, Vidya Gaikwad

**Affiliations:** 1 Obstetrics and Gynecology, Dr. D. Y. Patil Medical College, Hospital, and Research Centre, Dr. D. Y. Patil Vidyapeeth (Deemed to be University), Pune, IND

**Keywords:** polyp, endometrium, histopathology, hysteroscopy, abnormal uterine bleeding (aub)

## Abstract

Background: Abnormal uterine bleeding (AUB) is an important clinical entity, and its subtype, heavy menstrual bleeding (HMB), affects 14%-25% of women of reproductive age, potentially impairing their physical, emotional, social, and material quality of life. However, in addition to prior studies that supported the efficacy of hysteroscopy in identifying AUB, this study determined the overall pattern of abnormalities in AUB and correlated the diagnosis through hysteroscopy and histopathology. Additionally, a comparison of ultrasonography was done in this study. This study helps healthcare providers provide timely diagnosis and, thereby, timely interventions in treating different causes of AUB.

Methodology: This hospital-based prospective observational study was conducted at a tertiary care center, including women aged 20-60 admitted with complaints of AUB between September 2022 and June 2024. Participants were selected based on their willingness to participate in the study. A statistical analysis was performed using relevant descriptive statistics and plotting the frequency and percentage. Cohen's kappa was applied to ascertain significant associations and patterns within the dataset.

Results: The study included 47 women who were admitted to the hospital for diagnostic hysteroscopy. A majority of clinical symptoms presented as menorrhagia (17, 36.2%). The hemoglobin (g/dL) ranged from 5.9 to 14.7. Overall, on hysteroscopy, 37 (78.7%) women had a normal uterus. On hysteroscopy, 36 (76.6%) participants showed a normal cervical canal and cervix. In terms of histopathology, a majority of the study population (21, 44.7%) had proliferative endometrium. In terms of the correlation between hysteroscopy and histopathology, the two methods agreed in 83% of cases and disagreed in 17% of cases.

Conclusion: This study confirms that hysteroscopy is a crucial tool for assessing patients with AUB, particularly those with a thick endometrium, across all age groups. It does not replace other diagnostic methods; instead, it enhances them. Hysteroscopic-guided biopsy along with histopathology is now widely regarded as the most reliable method and *new gold standard* for assessing cases of AUB.

## Introduction

Abnormal uterine Bleeding (AUB) refers to any variation from regular menstruation in frequency, regularity, duration, and flow volume [1]. Abnormal endometrium is a common feature of gynecological practice for various clinical diseases. Heavy menstrual bleeding is among the most frequent symptoms [2]. The International Federation of Gynecology and Obstetrics (FIGO), an expert panel on menstrual diseases, suggested a categorization system (PALM-COEIN) for causes of AUB [1]. AUB is a common issue in primary care. Abnormal bleeding is a common occurrence among women in their reproductive years, affecting approximately 30% of them [3]. An extensive examination of the patient's medical history and a thorough physical assessment are necessary to evaluate the cause of AUB [4]. Comprehensive investigations are utilized to establish a differential diagnosis [5]. AUB's etiology and differential diagnosis are diverse and complicated [3].

AUB is most commonly tied to anovulatory menstrual periods, making teenagers and perimenopausal women most vulnerable. Approximately 20% of those affected are adolescents, while 50% are between the ages of 40 and 50 [3]. Ultrasound imaging is an affordable, non-invasive, and easy method of evaluating uterine disease. Therefore, it is recommended as the primary diagnostic method for investigating uterine diseases in women experiencing AUB [6]. While hysteroscopy, using an endoscope, is used to directly observe the uterine cavity through the cervix [7].

In AUB, hysteroscopy has almost completely replaced blind curettage [8]. Dilatation and curettage (D&C), a blind surgery approach, has been replaced by the *see and treat* strategy [9]. Hysteroscopy, which directly analyzes the uterine cavity, is a reliable approach for studying women with AUB. It is simple to conduct, generally available, and may accurately identify hyperplasia of the endometrium, endometrial polyps, and sub-mucosal myoma [10]. In the presence of organic lesions, hysteroscopy allows for the identification of the most likely source of uterine bleeding, raising the possibility that tissue obtained during a guided biopsy would produce an accurate histologic diagnosis [11].

Histopathology is completely diagnostic for endometrial hyperplasia and cancer [12]. Histopathology may supplement hysteroscopy, although the efficacy of hysteroscopy as a standalone treatment for AUB management has yet to be investigated. As a result, there is a pressing need, in comparing the effectiveness of hysteroscopy and histopathology, to determine the functionality of hysteroscopy as a screening technique for diagnosing the cause of AUB.

## Materials and methods

Study design and setting

This was a hospital-based prospective observational study performed at Dr. D. Y. Patil Medical College, Hospital and Research Centre, Dr. D. Y. Patil Vidyapeeth (Deemed to be University), Pune. The study period extended from September 2022 to June 2024. Before the commencement of the investigation, approval was obtained from the Institute’s Scientific and Ethics Committee (ethical committee clearance number: IESC/393/2022).

Inclusion and exclusion criteria

Women aged 20 to 60 who were admitted for diagnostic hysteroscopy typically presented with AUB that had persisted for more than six months or less than three months without a satisfactory response to treatment. Additionally, those experiencing severe symptoms were also candidates for this procedure. The indications for diagnostic hysteroscopy included cases where ultrasonography revealed a normal or slightly bulky uterus or the presence of any structural abnormalities or lesions within the uterine cavity. The study excluded individuals under the age of 20, those undergoing hysteroscopy for infertility, and women with pelvic inflammatory disease. Additionally, patients were excluded if they had hip joint pathology, suspected pregnancies, preexisting thyroid dysfunction, or coagulopathies identified in preoperative investigations. Women with significant renal, cardiac, or pulmonary conditions, whether or not accompanied by fluid overload, were also excluded.

Sample size

Considering that the study by Das et al. [13], "Correlation of Hysteroscopic and Histopathological Findings in Diagnosing AUB in a Tertiary Care Center," showed a proportion of cases with proliferative, normal, or atrophic endometrium at 58.9%, we calculated a sample size of 47 with a 95% confidence interval (CI), an acceptable difference of 15%, and an expected loss to follow-up of 10%. The software used was Winpepi 11.38.

Data collection and consent

During the pre-selective visit, the inclusion and exclusion criteria were applied, and a detailed history was taken, focusing on the nature and severity of vaginal bleeding and symptoms indicative of significant pathology. A comprehensive physical examination, including a local pelvic exam, was performed, and a pelvic ultrasound was obtained. Written and informed consent for surgery was secured from all patients. Nulliparous women or those with cervical stenosis were administered 400 mg of vaginal misoprostol, while others proceeded without it. Spinal anesthesia was provided by an anesthetist, and diagnostic hysteroscopy was conducted by an experienced examiner using a Bettocchi hysteroscope with a 2.9 mm telescope and a 30° angle view. The hysteroscope was connected to an LED light source via a Stryker 1288 HD endoscope camera system, and audiovisual recordings were captured in the operating theater. The hysteroscope was also linked to a Hamou endomat, maintaining pressure at 100 mmHg with a flow rate of 40-60 mL. Uterine cavity distension was achieved using saline solution (0.9%) at pressures between 200 and 120 mmHg, aiming for minimal effective pressure. The cavity was examined for polyps, masses, myomas, or polypoidal endometrium, and samples were collected via the operative channel using various instruments. E-books, atlases, and standard reference textbooks were used for comparison and clinical diagnosis, after which the samples were labeled, preserved in 10% formalin, and sent for histopathological analysis. Follow-up included reviewing the histopathology report, and data entry and statistical analysis were performed while ensuring patient confidentiality throughout the study.

Statistical analysis

Data entry was done utilizing MS Excel (Microsoft Corporation, Redmond, WA). A statistical analysis was performed using GraphPad Prism 10. Relevant descriptive statistics were analyzed and plotted as frequency and percentage. To ascertain significant associations and patterns within the dataset, Cohen’s kappa was applied with values ≤ 0 as indicating no agreement and 0.01-0.20 as none to slight, 0.21-0.40 as fair, 0.41-0.60 as moderate, 0.61-0.80 as substantial, and 0.81-1.00 as almost perfect agreement. 

## Results

**Figure 1:**
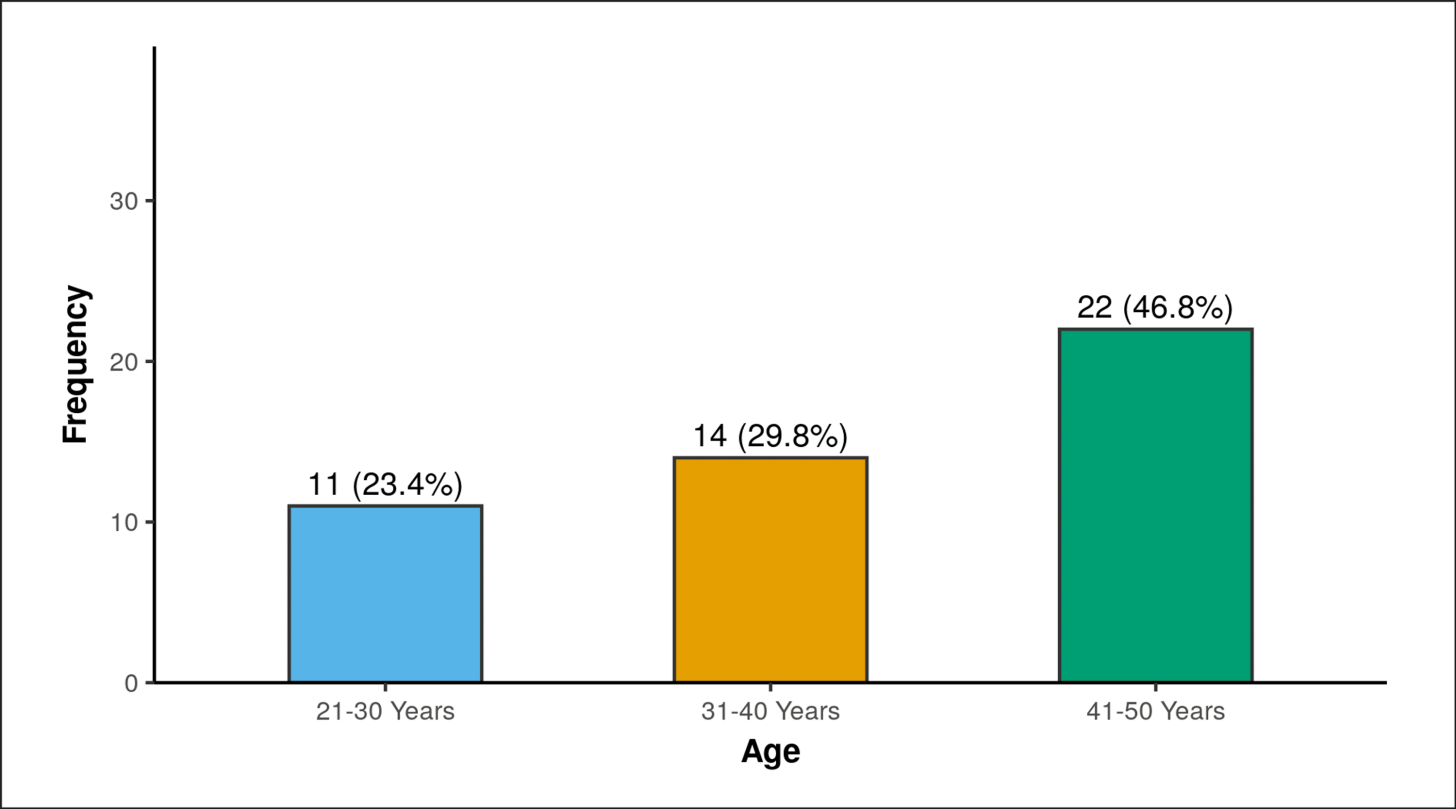
Distribution of age.

The study involved 47 women. Eleven (23.4%) of the participants were between the ages of 21 and 30 years, 14 (29.8%) of the women were between the ages of 31 and 40 years, and 22 (46.8%) of the women were between the ages of 41 and 50 years. The mean (SD) of age (years) was 37.55 (7.23). The median (IQR) was 40.00 (31.5-43). The age (years) ranged from 22 to 48 (Figure 1).

**Figure 2:**
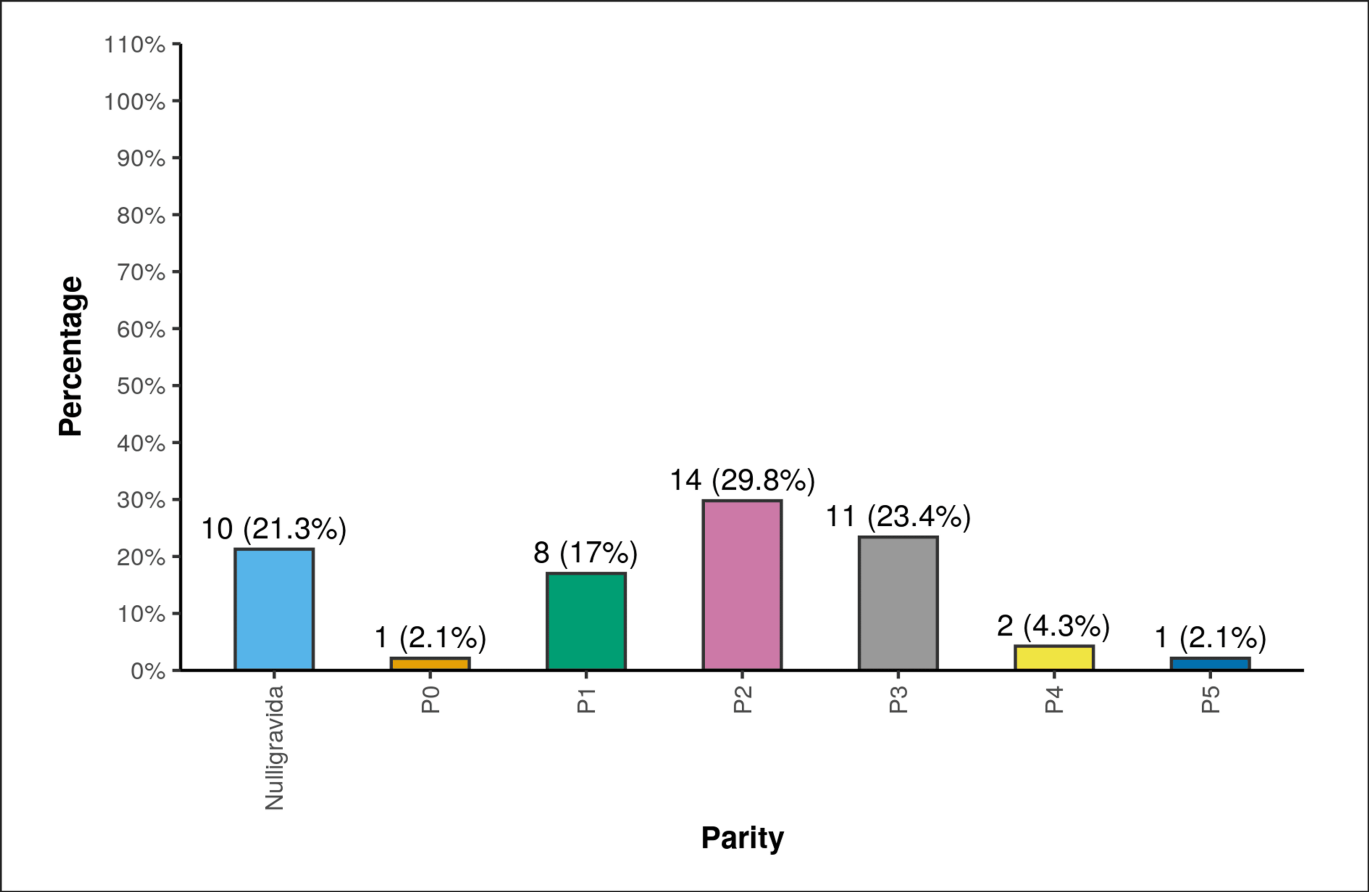
Distribution of parity. P, parity

In our study population, 11 (21.3%) of the participants were nulligravida and 8 (17%) of the participants had parity 1. Fourteen (29.8%) of the participants had parity 2. Eleven (23.4%) of the participants had parity 3. Two (4.3%) of the participants had parity 4. One (2.1%) of the them had parity 5 (Figure 2).

**Figure 3:**
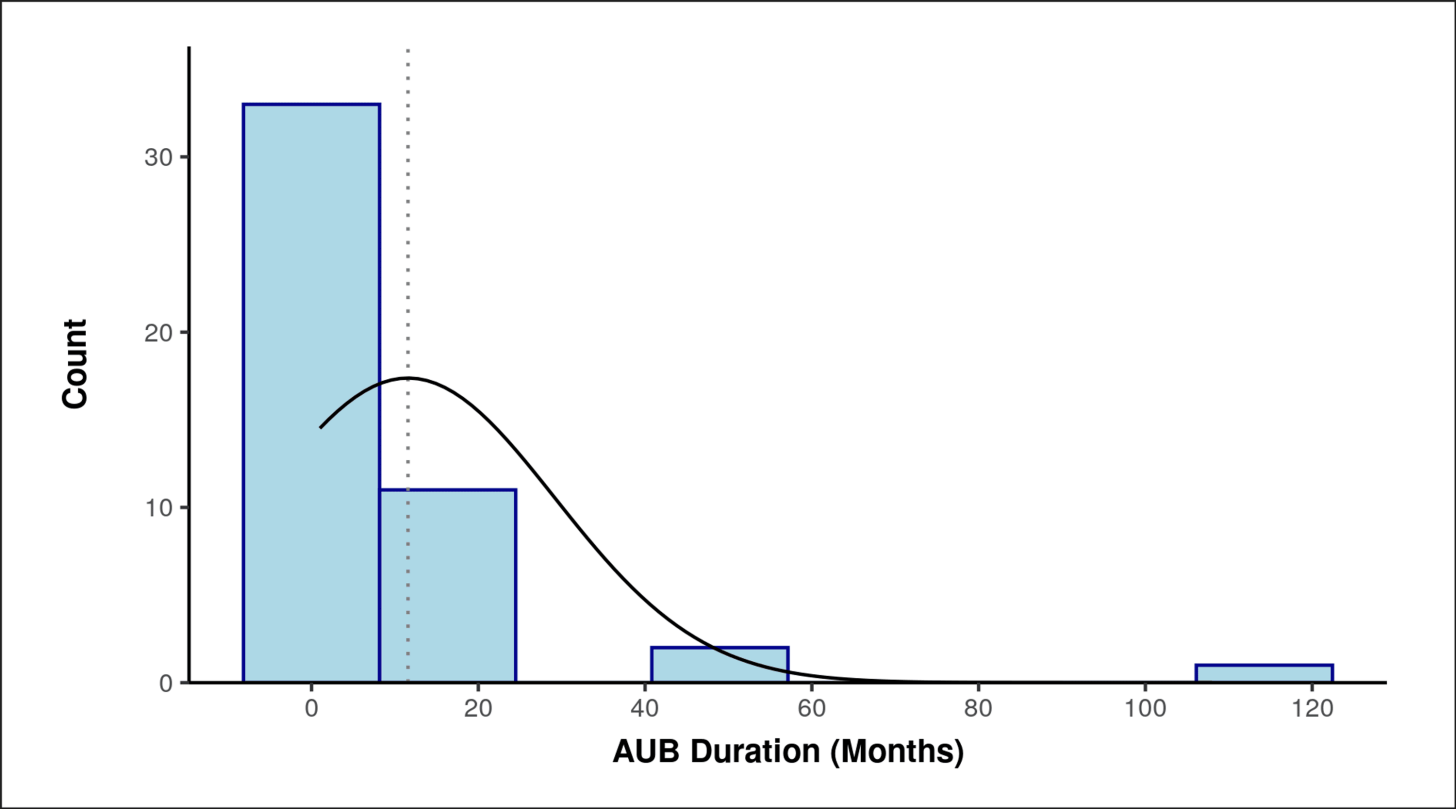
Distribution of the participants in terms of duration of AUB. AUB, abnormal uterine bleeding

The mean (SD) of AUB duration (months) was 11.57 (17.61). The median (IQR) of AUB duration (months) was 6.00 (4-12). The AUB duration (months) ranged from 1 to 108. The skewness of the data was 3.85, and it suggested that the data was positively skewed, thus suggesting it was not normally distributed. The following histogram was plotted with the actual counts, and an ideal bell-shaped curve was superimposed on top for reference (Figure 3). 

In our study, the majority of participants comprising of 17 (36.2%) had heavy, prolonged bleeding. Six (12.8%) of the participants had intermenstrual bleeding, irregular cycles. Seven (14.9%) of the participants had infrequent cycles. Four participants (8.5%) experienced heavy bleeding with frequent cycles. Two participants (4.3%) had frequent cycles. Eleven participants (23.4%) had heavy intermenstrual bleeding with irregular cycles (Table 1).

**Table 1:** Distribution of the participants in terms of the pattern of AUB. AUB, abnormal uterine bleeding; n, frequency; %, percentage; CI, confidence interval

AUB pattern	Old terminology	*n* (%)	95% CI
Heavy, prolonged bleeding	Menorrhagia	17 (36.2%)	23.1%-51.5%
Intermenstrual bleeding, irregular cycles	Metrorrhagia	6 (12.8%)	5.3%-26.4%
Infrequent cycles	Oligomenorrhoea	7 (14.9%)	6.7%-28.9%
Heavy bleeding, frequent cycle	Polymenorrhagia	4 (8.5%)	2.8%-21.3%
Frequent cycles	Polymenorrhea	2 (4.3%)	0.7%-15.7%
Heavy, intermenstrual bleeding, irregular cycles	Menometrorrhagia	11 (23.4%)	12.8%-38.4%

**Figure 4:**
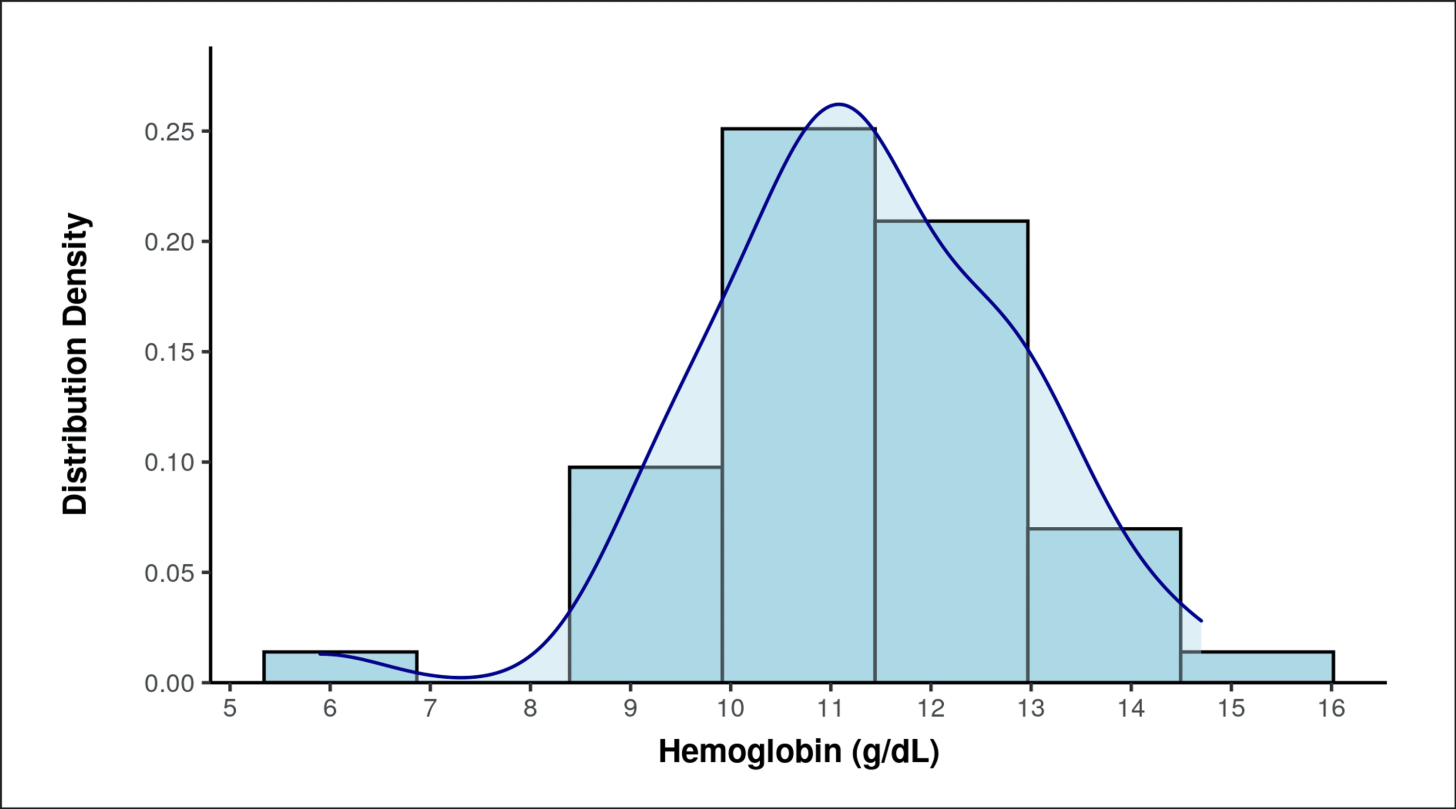
The distribution of hemoglobin among the participants.

The mean (SD) hemoglobin level (g/dL) was 11.28 (1.57), with a range from 5.9 to 14.7 g/dL (Figure 4).

Upon examining the ultrasonography findings in women with AUB, 19 participants (40.4%) had normal ultrasonography results. Ten participants (21.3%) had a bulky uterus, 9 participants (19.1%) had a fibroid, and 5 participants (10.6%) had a polyp. Additionally, 2 participants (4.3%) had endometrial hyperplasia, 1 participant (2.1%) had adenomyosis, and 1 participant (2.1%) had a uterine septum (Table 2). Among the 9 participants who had fibroids, 3 had posterior wall submucosal fibroids, 2 had anterior wall submucosal fibroids, and 4 had multiple fibroids along the lateral walls of the uterus.

**Table 2:** Distribution of the participants in terms of ultrasonography. USG, ultrasonography; n, frequency; %, percentage

USG	Yes, *n* (%)	No, *n* (%)
Normal	19 (40.4%)	28 (59.6%)
Bulky	10 (21.3%)	37 (78.7%)
Fibroid	9 (19.1%)	38 (80.9%)
Polyp	5 (10.6%)	42 (89.4%)
Endometrial hyperplasia	2 (4.3%)	45 (95.7%)
Adenomyosis	1 (2.1%)	46 (97.9%)
Septum noted	1 (2.1%)	46 (97.9%)

**Figure 5:**
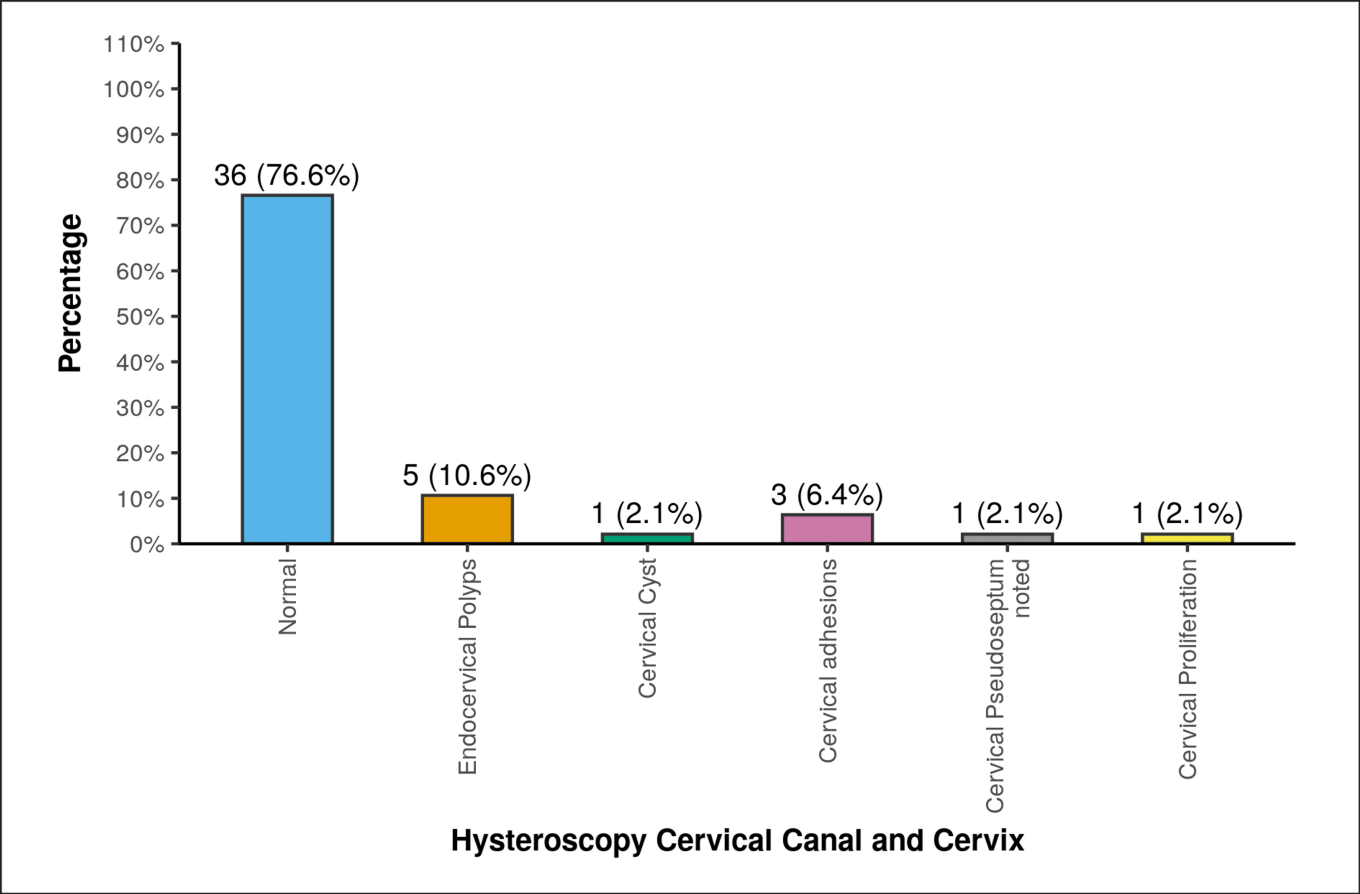
Distribution of participants in terms of hysteroscopy of the cervical canal and the cervix.

A majority of the participants (36, 76.6%) in the current study had normal cervical canals and cervixes on hysteroscopy. Five participants (10.6%) had endocervical polyps, 1 participant (2.1%) had cervical cysts, and 3 participants (6.4%) had cervical adhesions. Additionally, 1 participant (2.1%) had a cervical pseudoseptum, and 1 participant (2.1%) had cervical proliferation (Figure 5).

Out of the participants, 18 (38.3%) had a normal endometrium during hysteroscopy. Endometrial polyps, described as small, soft, oval, pedunculated, and smooth growths within the uterine cavity, were identified in 8 participants (17.0%). Six participants (12.8%) had a polypoidal endometrium, characterized by a thickened and polypoidal appearance. Additionally, 4 participants (8.5%) exhibited a proliferative endometrium, which appeared pink, uniformly smooth, and thin, while 3 participants (6.4%) had endometritis, identified by focal or diffuse hyperemia, stromal edema, the presence of micropolyps, and the typical strawberry aspect. Adenomyosis, which was identified by an irregular endometrial vascular distribution pattern, both throughout the proliferative and secretory phases was noted in 4 participants (8.5%), while fibroids were noted in 2 participants (4.3%), observed as a white-colored bulge, round in shape, with a smooth surface, on hysteroscopy. Finally, 2 participants (4.3%) were found to have endometrial adhesions, visualized as either thin or thick stretchy bands of white scar tissue (Table 3).

**Table 3:** Summary of the hysteroscopy findings of the endometrium. n, frequency; %, percentage

Hysteroscopy findings of endometrium	Yes, *n* (%)	No, *n* (%)
Normal	18 (38.3%)	29 (61.7%)
Polyps	8 (17.0%)	39 (83.0%)
Polypoid	6 (12.8%)	41 (87.2%)
Proliferative	4 (8.5%)	43 (91.5%)
Endometritis	3 (6.4%)	44 (93.6%)
Adenomyosis	4 (8.5%)	43 (91.5%)
Fibroid	2 (4.3%)	45 (95.7%)
Adhesions	2 (4.3%)	45 (95.7%)

In our study, 21 participants (44.7%) had histopathology showing proliferative endometrium. Thirteen participants (27.7%) had histopathology indicating secretory endometrium. Two participants (4.3%) had chronic endometritis on histopathological examination (HPE). One participant (2.1%) had atrophic endometrium, and another participant (2.1%) had adenomyosis. Additionally, 2 participants (4.3%) had leiomyoma, 2 participants (4.3%) had endometrial hyperplasia, and 6 participants (12.8%) had endometrial polyps (Table 4).

**Table 4:** Distribution of participants in terms of histopathology examination of the endometrium. HPE, histopathology examination; n, frequency; %, percentage; CI, confidence interval

HPE of endometrium	*n* (%)	95% CI
Proliferative endometrium	21 (44.7%)	30.5%-59.8%
Secretory endometrium	13 (27.7%)	16.1%-42.9%
Chronic endometritis	2 (4.3%)	0.7%-15.7%
Atrophic endometrium	1 (2.1%)	0.1%-12.7%
Adenomyosis	1 (2.1%)	0.1%-12.7%
Leiomyoma	2 (4.3%)	0.7%-15.7%
Hyperplasia	1 (2.1%)	0.1%-12.7%
Polyp	6 (12.8%)	5.3%-26.4%

A test for agreement was done. In the following table, the cells that are diagonally in bold represent cases where both the methods agreed. The two methods agreed in 83.0% of the cases and disagreed in 17.0% of the cases (Table 5).

**Table 5:** Comparison between hysteroscopy and histopathology findings in AUB. AUB, abnormal uterine bleeding; HPE, histopathology examination; n, frequency; %, percentage

Diagnosis		Diagnosis (HPE)								Cohen's Kappa	
		Endocervical polyp, *n *(%)	Endometrial polyp, *n* (%)	Adenomyosis, *n* (%)	Leiomyoma, *n* (%)	Endometrial hyperplasia, *n* (%)	Normal, *n* (%)	Endometritis, *n* (%)	Total, *n* (%)	k	*P*-value
Diagnosis (Hysteroscopy)	Endocervical polyp, *n *(%)	4 (8.5%)	0 (0.0%)	0 (0.0%)	0 (0.0%)	0 (0.0%)	0 (0.0%)	0 (0.0%)	4 (8.5%)	0.774	<0.001
	Endometrial polyp, *n* (%)	0 (0.0%)	14 (29.8%)	0 (0.0%)	0 (0.0%)	0 (0.0%)	0 (0.0%)	0 (0.0%)	14 (29.8%)		
	Adenomyosis, *n* (%)	0 (0.0%)	0 (0.0%)	2 (4.3%)	0 (0.0%)	0 (0.0%)	3 (6.4%)	1 (2.1%)	6 (12.8%)		
	Leiomyoma, *n* (%)	0 (0.0%)	1 (2.1%)	0 (0.0%)	2 (4.3%)	0 (0.0%)	0 (0.0%)	0 (0.0%)	3 (6.4%)		
	Endometrial hyperplasia, *n* (%)	0 (0.0%)	0 (0.0%)	0 (0.0%)	0 (0.0%)	0 (0.0%)	2 (4.3%)	0 (0.0%)	2 (4.3%)		
	Normal,* n* (%)	0 (0.0%)	0 (0.0%)	1 (2.1%)	0 (0.0%)	0 (0.0%)	14 (29.8%)	0 (0.0%)	15 (31.9%)		
	Endometritis, *n* (%)	0 (0.0%)	0 (0.0%)	0 (0.0%)	0 (0.0%)	0 (0.0%)	0 (0.0%)	3 (6.4%)	3 (6.4%)		
	Total, *n *(%)	4 (8.5%)	15 (31.9%)	3 (6.4%)	2 (4.3%)	0 (0.0%)	19 (40.4%)	4 (8.5%)	47 (100.0%)		

There was a statistically significant agreement between the two variables (Cohen's kappa = 0.774, *P* < 0.001). As per statistical convention, a kappa value of 0.774 denotes substantial agreement, as it lies between 0.61 and 0.80 (Table 6). 

**Table 6:** Correlation between hysteroscopy and histopathology findings in AUB. AUB, abnormal uterine bleeding

Categories	Kappa	*P*-value
Endocervical polyp	1.000	<0.001
Endometrial polyp	0.950	<0.001
Adenomyosis	0.386	<0.001
Leiomyoma	0.789	<0.001
Endometrial hyperplasia	-0.022	<0.001
Normal	0.724	<0.001
Endometritis	0.846	<0.001

The sensitivity, specificity, positive predictive value (PPV), negative predictive value (NPV), and diagnostic accuracy of hysteroscopy for detecting endometrial polyps were 100%, 93.33%, 96.97%, 100%, and 97.87%; for adenomyosis were 93.18%, 100%, 100%, 50%, and 93.62%; for leiomyoma were 97.78%, 100%, 100%, 66.67%, and 97.87%; for endometrial hyperplasia were 100%, 93.33%, 96.97%, 100%, and 97.87%; for normal endometrium were 87.5%, 100%, 100%, 78.95%, and 91.49%; and for endometritis were 97.73%, 100%, 100%, 75%, and 97.87%, respectively (Table 7).

**Table 7:** Summary of parameters related to hysteroscopy and histopathology findings. PPV, positive predictive value; NPV, negative predictive value; CI, confidence interval

Condition	Sensitivity (95% CI)	Specificity (95% CI)	PPV (95% CI)	NPV (95% CI)	Diagnostic accuracy (95% CI)
Endometrial polyp	100% (89.28-100)	93.33% (70.18-98.81)	96.97% (84.68-99.46)	100% (78.47-100)	97.87% (88.89-99.62)
Adenomyosis	93.18% (81.77-97.65)	100% (43.85-100)	100% (91.43-100)	50% (18.76-81.42)	93.62% (82.84-97.81)
Leiomyoma	97.78% (88.43-99.61)	100% (34.24-100)	100% (91.97-100)	66.67% (20.77-93.85)	97.87% (88.89-99.62)
Endometrial hyperplasia	100% (89.28-100)	93.33% (70.18-98.81)	96.97% (84.68-99.46)	100% (78.47-100)	97.87% (88.89-99.62)
Normal endometrium	87.5% (71.93-95.03)	100% (79.61-100)	100% (87.94-100)	78.95% (56.67-91.49)	91.49% (80.07-96.64)
Endometritis	97.73% (88.19-99.6)	100% (43.85-100)	100% (91.8-100)	75% (30.06-95.44)	97.87% (88.89-99.62)

## Discussion

Hysteroscopic examination provides a precise prediction of endometrial lesions and precisely defines the different characteristics of the endometrium, using the same terminology as pathologists. This technology enables the correlation between hysteroscopic observations and histology data, which was useful to this study. The present study revealed that 22 (46.8%) of the sample population fell within the age range of 41-50 years, indicating a significant majority. Taneja et al. observed that almost two-thirds of the entire patient population were 36 years old or older, a finding that aligns with our own study [14]. Panda et al. discovered that most patients fell between the age range of 35 and 45 years [15]. Similarly, the study conducted by Guin et al. found that the average age of the patients in their research was 39.74 years [16]. This age range aligns with the aforementioned group. Thus, most cases have occurred in the perimenopausal age group. Needless to say, hormonal imbalances and abnormal uterine pathologies are more common in the older age group. Hysteroscopy, therefore, is a minimally invasive tool at this stage of life and can aid in correct sampling along with histopathology correlation. Perimenopausal AUB is the most common presentation, and a correct diagnosis can also be a therapeutic modality.

In our study, 10 (21.3%) were nulligravida, while 14 (29.8%) were parity 2. According to Guin et al. [16], the average parity was 3.1. A study conducted by Jyotsana et al. [17] revealed that 54.7% of participants were multiparous, which significantly aligns with the findings of our study. With advancing parity, the AUB incidence can be easily diagnosed with hysteroscopy as the cervical oz is easily dilated, sometimes even with the lack of misoprostol. In our study, the mean duration of AUB was 11.57 (17.61), or almost 12 months. The median duration of AUB was six months (4-12). Singh et al. [18] reported in their study that the symptoms lasted for a mean of 7.3 months. The majority of these women sought advice from gynecologists within one year of experiencing symptoms. 

In the current study, the mean hemoglobin (g/dL) was 11.28 (1.57), and the hemoglobin ranged from 5.9 to 14.7. According to the study by Das et al. [13], a significant proportion of women (50.7%) had mild anemia. Most of the patients with AUB tend to have anemia due to increased blood loss for a prolonged period outside of the normal menstrual cycle. The mean ET was 8.66 mm (3.99). The median ET was 7.00 mm. The ET ranged from 4 to 23 mm. Das et al. [13] found that 68.67% of women had endometrial thickness on TVS that ranged between 5 and 10 mm. Only 10.7% had ET < 5 mm, whereas 20.7% had ET > 10 mm [5]. In this investigation, the maximum ET observed on TVS was 5 to 8 mm. Six patients revealed underwent hysteroscopy that confirmed the USG showing an ET greater than 12 mm, and a biopsy of the endometrium was sent for examination with suspicion of malignancy. For perimenopausal women, ET was commonly around 8 mm according to most research, and the current study found a similar result. 

**Figure 6:**
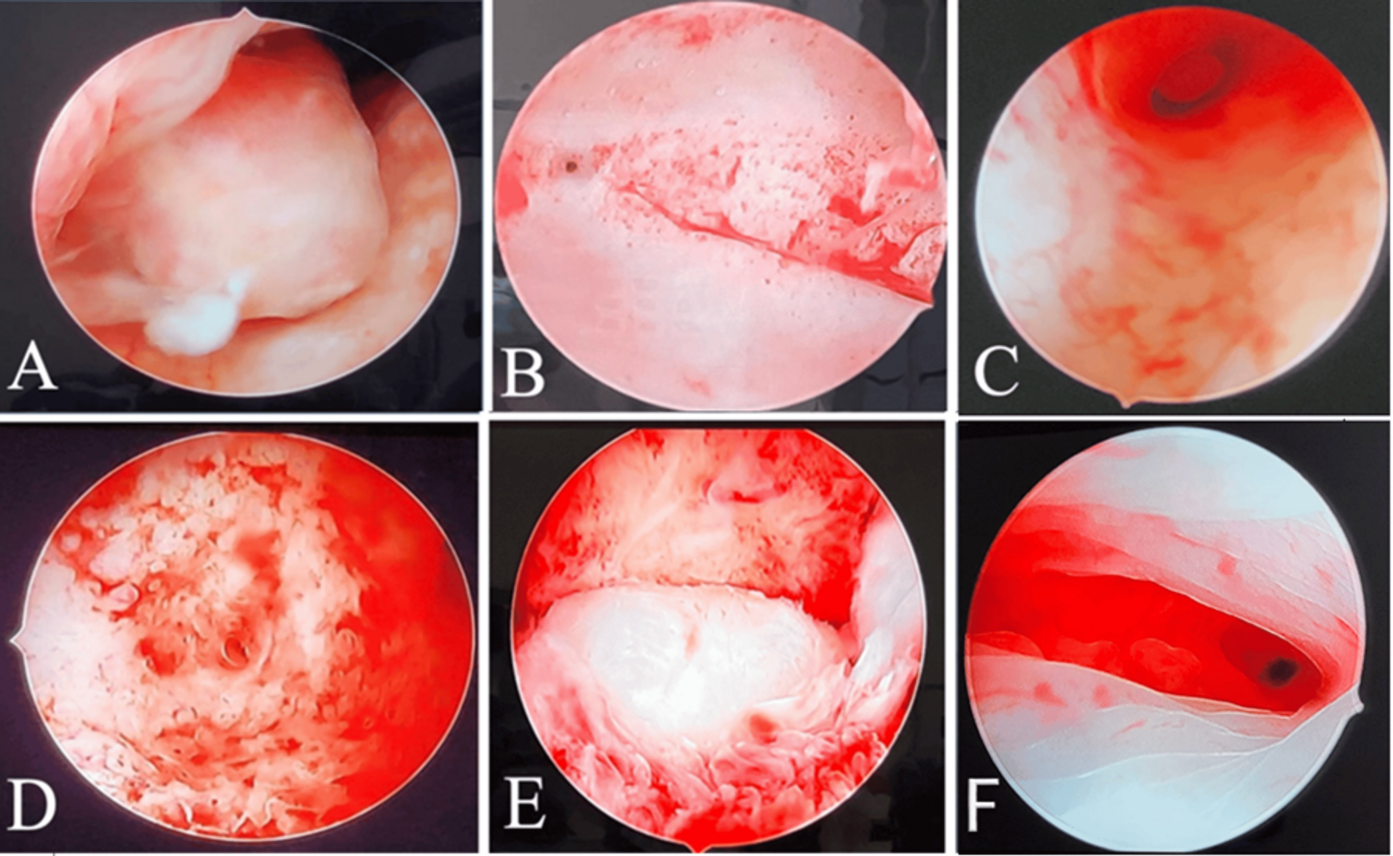
Hysteroscopy image of (A) endometrial polyp, (B) proliferative endometrium, (C) endometritis, (D) adenomyosis, (E) submucosal fibroid seen bulging through the endometrium, and (F) secretory endometrium.

In the current study, after performing hysteroscopy, it was revealed that 20 (42.6%) participants had a normal uterus, while 27 (57.4%) had an abnormal finding. In our research, 8 (17.0%) of the endometrium included polyps (Figure 6A). This was a particularly frequent finding in our research when examining the endometrium. Taneja et al. [19] found that out of 70 instances of menorrhagia diagnosed with hysteroscopy, six were endometrial polyps. Similarly, Naik et al. [20] noted that polyps were the most common finding on hysteroscopy in 26.8% of cases. Singh et al. [18] found endometrial polyps in 18.6% of cases, which is similar to our findings. This was in contrast to Kumar et al.'s study. [21], in which endometrial hyperplasia (42%) was the most common aberrant result, followed by polypoidal endometrium with mucosal polyps (22%).

In our study, 6 (12.8%) had a polypoid endometrium, which was observed when the endometrium appeared to be thickened and polypoidal. Naik et al. [20] noted in their study that 5.1% of patients had a polypoidal hyperplastic endometrium. In the study by Singh et al. [18], 66% of patients were seen with polypoidal hyperplastic endometrium. In the current study, 8.5% constituted of proliferative endometrium. Valson et al. [22] observed that 12% had hyperplastic endometrium in a study conducted by them. In the study by Sunita et al. [23], 6% of cases had proliferative endometrium (Figure 6B).

In the current study, 3 (6.4%) showed endometritis (Figure 6C). In the study by Kumar et al. [21], it was observed that 2% of cases had shown endometritis. In another study by Valson et al. [22], 4% had endometritis. Sunitha et al. [23] found that in their study, there were 2% cases of endometritis. In our study, 4 (8.5%) had adenomyosis (Figure 6D), while 2 (4.3%) had fibroids (Figure 6E). In the study by Naik et al. [20], 4.85% had a submucosal leiomyoma on hysteroscopy, seen as a protrusion in the wall of the uterus. Singh et al. [18] found in the research that they had done, that out of 150 patients, 15% showed myoma indenting the uterine cavity on hysteroscopy. Kumar et al. [21], in their study, observed that there were 4% cases of submucous myomas on hysteroscopy. Sunitha et al. [23] observed in their study that there were 4% of submucous myomas, which were all similar to our study.

In the current study, 2 (4.3%) had endometrial adhesions, also called uterine synechiae. In the study by Naik et al. [20], they noticed adhesions in 0.85% of cases. Kumar et al. [21] observed that 2% of patients had uterine synechiae on hysteroscopy. Hysteroscopy shows an orange, undulating, and thick endometrium appearing to be a secretory endometrium (Figure 6F). In terms of histopathology in the current study, 21 (44.7%) of the study population had proliferative endometrium. Thirteen (27.7%) of them had secretory endometrium. Among the abnormal pathologies, 6 (12.8%) of the participants had an endometrial polyp. 2 (4.3%) of them had chronic endometritis, 1 (2.1%) had an atrophic endometrium, 1 (2.1%) had adenomyosis on HPE, 2 (4.3%) had leiomyoma, and 1 (2.1%) had endometrial hyperplasia. Similar to our results, Subburaj et al. [24] discovered that proliferative endometrium (34%), which is the most prevalent finding according to histopathological diagnosis, was followed by polyps (28%). In a study similar to the current one, Taneja et al. [19] reported that, upon histopathological examination, 52.5% of cases had proliferative endometrium, 11.7% had secretory endometrium, being the second most common finding, 2.5% had atrophic endometrium, and 7.5% had chronic endometritis. In their study, Sunitha et al. [23] observed that histopathology accurately identified all cases of endometrial hyperplasia, which accounted for 20% of cases, atrophic endometrium for 8% of cases, and endometritis for 2%. 

The following differences were observed in the diagnosis, four patients who had endocervical polyps were diagnosed on both hysteroscopy and histopathology with 100% accuracy. Out of 15 patients diagnosed by histopathology with endometrial polyps, 14 were observed through hysteroscopy, and one was missed out. Adenomyosis is a difficult finding to make through histopathology, which was seen in 6 (12.8%) of cases through hysteroscopy but diagnosed in 3 (6.4%) of cases by histopathology. Out of 3 (6.4%) cases where fibroid was visualized, 2 (4.3%) cases were confirmed as leiomyoma, and another case turned out to be an endometrial polyp. Out of the 18 (38.3%)% cases thought to be normal endometrium as visualized through hysteroscopy, 3 (6.4%) cases were diagnosed by histopathology as adenomyosis, and 2 (4.3%) cases were endometrial hyperplasia. Endometritis was diagnosed through hysteroscopy in 3 out of 4 cases with a 75% accuracy.

The current investigation looks at the relationship between histopathology and hysteroscopy. In 83.0% of the situations, both approaches showed concurrence, whereas in 17.0% of the cases, they showed discordance. According to the Stuart-Maxwell test, the overall diagnosis difference was not statistically significant. There was a statistically significant correlation among the two variables, as indicated by a Cohen's kappa value of 0.774 (*P* < 0.001). The kappa score was 0.774, indicating significant agreement. Valson et al. [22] did a study to examine the detection of abnormal pathology using hysteroscopy and histology. Of the outcomes, 96% were comparable. The specificity, sensitivity, positive predictive value (PPV), and negative predictive value (NPV) were 90.9%, 53.8%, 77.8%, and 90.9%, respectively.

The hysteroscopy test detects endometrial polyps with 100% sensitivity, 93.33% specificity, 96.97% PPV, 100% NPV, and 97.87% diagnostic accuracy. Patil et al. [25] showed that the hysteroscopic diagnosis accuracy for hyperplasia was 72%. Hysteroscopy for hyperplasia had 75% sensitivity, 92.5% specificity, a PPV of 71.4%, and an NPV of 93.67%. Loverro et al. [26] reported the following findings for endometrial hyperplasia: 98% sensitivity, 95% specificity, 63% PPV, and 99% NPV. Endometrial polyps were detected by hysteroscopy in the study by Firdous et al. [9], with a 100% sensitivity, 94.1% specificity, 88% PPV, and 100% NPV. In line with our findings, the diagnosis accuracy was likewise reported at 100%. 

Hysteroscopy accurately detects adenomyosis with 93.18% sensitivity, 100% specificity, 100% PPV, 50% NPV, and 93.62% diagnostic accuracy. Although there is no consensus on the diagnostic hysteroscopic criteria for adenomyosis, it appears that hysteroscopy can play an essential role in diagnosing this pathology. The sensitivity, specificity, PPV, NPV, and diagnostic accuracy of hysteroscopy for detecting leiomyoma were 97.78%, 100%, 66.67%, and 97.87%, respectively. Patil et al. [25] found that hysteroscopy had 100% diagnostic accuracy for submucous fibroids. The sensitivity, specificity, PPV, and NPV of hysteroscopy for fibroids compared to histology were 100%, 89.89%, 9.09%, and 100%, respectively, supporting our findings.

The sensitivity, specificity, PPV, NPV, and diagnostic accuracy of hysteroscopy for detecting endometrial hyperplasia were 100%, 93.33%, 96.97%, 100%, and 97.87%. Patil et al. [25] reported a 72% hysteroscopic diagnosis accuracy for hyperplasia. Therefore, the sensitivity, specificity, PPV, and NPV of hysteroscopy for hyperplasia were 75%, 92.5%, 71.4%, and 93.67%, respectively. Loverro et al. [26] reported a sensitivity, specificity, PPV, and NPV of 98%, 95%, 63%, and 99% for endometrial hyperplasia, respectively. Firdous et al. [11] found that hysteroscopy had a sensitivity, specificity, PPV, and NPV of 92.6%, 97.3%, 92.6%, and 97.3% for identifying hyperplasia, respectively.

The sensitivity, specificity, PPV, NPV, and diagnostic accuracy of hysteroscopy for detecting normal endometrium were 87.5%, 100%, 78.95%, and 91.49%, respectively. Firdous et al. [9] found that the specificity, sensitivity, PPV, and NPV of detecting normal endometrium by hysteroscopy were 83.9, 93.2, 82, and 94%, respectively, with a diagnostic accuracy of 88%, which was comparable to our study. Hysteroscopy detected endometritis with 100% specificity, 97.73% sensitivity, 100% PPV, 75% NPV, and 97.87% diagnostic accuracy. Our findings were supported by Singh et al.'s [14] study, which discovered that hysteroscopy had a 100% diagnostic accuracy for tuberculous endometritis. As a result, hysteroscopy for tuberculous endometritis has 100% sensitivity, specificity, PPV, and NPV when compared to histology. In a study conducted by Sunitha et al. [23], it was found that endometrial histopathology failed to detect four cases of endometrial polyps as well as one case of submucous myoma. Histopathology accurately identified all instances of endometrial hyperplasia (20%), atrophic endometrium (8%), and endometritis (2%). The study found that hysteroscopy, dilatation, and curettage yielded consistent results in 82% of patients. Hysteroscopy provided additional information compared to curettage in 12% of patients, whereas curettage provided more details than hysteroscopy in 6% of patients. In their study, Subburaj et al. [24] evaluated the statistical significance of hysteroscopic findings and histopathology findings in the participants. They concluded that among those who reported hyperplasia, most of the histopathological findings were confirmatory of simple hyperplasia (50%), while 33.3% showed proliferative endometrium. Furthermore, histology confirmed the presence of lesions in 70% of those who were diagnosed with polyps during hysteroscopy. The findings exhibited statistical significance (chi-square = 44.7, *P*-value < 0.0001), consistent with other studies. Therefore, it can be asserted that hysteroscopy has the crucial benefit of providing direct vision of any abnormalities within the uterine cavity. It serves as a supplementary tool to other diagnostic approaches, rather than replacing them. 

Limitations

The sample size was small, which limited the ability to determine individual pathology. Consequently, this ongoing study requires a larger sample size to draw more robust conclusions. Additionally, hysteroscopy was performed by a single observer, while the histopathological diagnosis was conducted by multiple pathologists.

## Conclusions

This research discusses the reliability of hysteroscopy as a precise method for diagnosing individuals with AUB, particularly when there are abnormalities in the endometrial cavity. In our study, findings can be summarized in terms of the average age group being 37 years. The majority were of parity 2, and a majority of clinical symptoms presented as either menorrhagia (36.2%) or menometrorrhagia (23.4), while 70.2% of them had irregular menstrual cycles, 61.7% had dysmenorrhea, 10.6% had cervical polyps, and even a cervical pseudoseptum could be detected, and 44.7% had a proliferative endometrium. It also has a satisfactory level of sensitivity, specificity, PPV, and NPV. Thus, a statistically significant agreement between hysteroscopy and histopathology was established in evaluating the cause of AUB. Moreover, hysteroscopy findings are promptly available and are an uncomplicated, expeditious, and cost-effective procedure that is widely embraced by patients and holds significant promise in the field of gynecology.
